# Dose-finding study and pharmacogenomic analysis of fixed-rate infusion of gemcitabine, irinotecan and bevacizumab in pretreated metastatic colorectal cancer patients

**DOI:** 10.1038/sj.bjc.6605908

**Published:** 2010-10-12

**Authors:** A Abajo, J Rodriguez, N Bitarte, R Zarate, V Boni, M Ponz, A Chopitea, E Bandres, J Garcia-Foncillas

**Affiliations:** 1 Laboratory of Pharmacogenomics, Division of Oncology, Center for Applied Medical Research (CIMA), University of Navarra, Pamplona, Spain; 2 Department of Oncology, University Clinic of Navarra, University of Navarra, Pamplona, Spain

**Keywords:** gemcitabine, irinotecan, bevacizumab, metastatic colorectal cancer, dose finding, VEGF

## Abstract

**Background:**

To determine the dose-limiting toxicity (DLT), maximum tolerated dose, recommended dose (RD) and preliminary evidence of activity of escalating doses of irinotecan (CPT-11) fixed-dose-rate infusional gemcitabine (FDR-GMB) and bevacizumab in pretreated metastatic colorectal cancer (mCRC) patients. Pharmacogenomic analysis was performed to investigate the association between VEGF single-nucleotide polymorphisms and clinical outcome.

**Patients and methods:**

A total of 89 mCRC patients were recruited in a two-step study design; 28 were included in the dose-finding study and 59 in the pharmacogenomic analysis. The FDR-GMB of 1000 mg m^–2^, bevacizumab 5 mg kg^–1^ and CPT-11 doses ranging from 100 to 160 mg m^–2^ were explored. The VEGF protein serum levels were quantified by EIA. Allelic discrimination was performed to genotype polymorphisms in the *VEGF* gene.

**Results:**

CPT-11 RD was 150 mg m^–2^. Diarrhoea and neutropenia were the DLT. After a median follow-up of 42 months, the median time to progression (TTP) and overall survival were 5.2 and 19.9 months, respectively. VEGF levels were significantly correlated with VEGF-2578AA and VEGF-460CC genotypes, and a trend was observed with VEGF+405GG genotype. The presence of any of these genotypes correlated with a longer median TTP (8.8 *vs* 4.5 months, *P*=0.04).

**Conclusion:**

The triplet combination tested in this study is effective and well tolerated. A possible predictive role for VEGF gene polymorphisms and baseline VEGF circulating levels is suggested.

To date, most patients with relapsed metastatic colorectal cancer (mCRC) receive second or further lines of systemic therapy (Rothenberg, 2004). Overall response rate in the range of 15–25% and survival times around 12 months have been reported, with varying degrees of improved outcome (Maindrault-Goebel *et al*, 1999; Tournigand *et al*, 2004). Irinotecan-based regimens are commonly used after failure of first-line oxaliplatin/fluoropyrimidine combinations (Tournigand *et al*, 2004). Although bevacizumab uniformly enhanced the cytotoxic antitumour effect of chemotherapy in the first-line setting of mCRC (Kabbinavar *et al*, 2003; Hurwitz *et al*, 2004), at the time we initiated this study, a limited efficacy had been reported with this agent in pretreated mCRC patients, possibly due to the low expected activity of the chemotherapy component (Emmanoulides *et al*, 2004). On these basis, and taking into account the modest outcome achieved with second-line FOLFIRI (Tournigand *et al*, 2004), we hypothesised whether the addition of bevacizumab to an irinotecan-based schedule without 5-fluorouracil may represent a promising strategy.

Gemcitabine (GMB), a difluorinated analogue of deoxycytidine, exerts its antitumour activity through inhibition of ribonucleotide reductase and DNA synthesis (Plunkett *et al*, 1995). Although prolonged exposure of this agent appeared to have superior activity when compared with bolus administration in murine colon tumours (Kornmann *et al*, 2000), several phase I/II trials of single-agent GMB had demonstrated minimal activity in mCRC patients (Moore *et al*, 1992; Mani *et al*, 1998; Lonardi *et al*, 2004). However clinical outcomes remarkably improve when GMB is used in combination regimens. In fact, a growing body of evidence suggests that GMB synergistically interacts with some of the most widely used agents in mCRC, including fluoropyrimidines (Correale *et al*, 2003) and oxaliplatin (Faivre *et al*, 1999). In addition, *in vitro* blockade of VEGF-receptor activation has proved to enhance the efficacy of GMB (Solorzano *et al*, 2001).

A synergistic sequence-dependent interaction of GMB and SN-38 has also been found in preclinical models, as the incorporation of GMB into DNA enhances campothecin-induced topo-1 cleavage complexes (Pourquier *et al*, 2002). Indeed, in colon cancer-derived cell lines, GMB was shown to induce the expression of all topoisomerase enzymes and cytotoxicity was more relevant when cells lines were treated with GMB and topoisomerase I posions within a short period of time (Richter *et al*, 2006).

This preclinical background has prompted the design of clinical studies with GMB-based combinations, mainly oxaliplatin and fluoropyrimidines, in pretreated mCRC patients (Correale *et al*, 2004), with interesting tumour growth control rates and a favourable toxicity profile.

The *VEGF* gene expression is upregulated in colorectal cancer and can be predictive of invasiveness, metastases, recurrence and prognosis (George *et al*, 2001). Numerous single-nucleotide polymorphisms (SNPs) in the promoter, 5′- and 3′-untranslated regions have been described (Brogan *et al*, 1999), although their predictive value regarding bevacizumab efficacy in mCRC remains to be determined.

On the basis of these considerations, we initiated this pilot study with a double aim; first, to determine the dose-limiting toxicity (DLT), the recommended dose (RD) and preliminary evidence of activity of this triplet combination. The second objective was to explore the association of baseline VEGF circulating levels, *VEGF* gene SNPs and clinical outcome.

## Patients and methods

### Eligibility

Eligibility criteria included age >18 years and a histologically confirmed mCRC progressed after one prior oxaliplatin/fluoropyrimidine-based chemotherapy regimen for metastatic disease. Pre-trial disease progression was radiologically confirmed and independently reviewed. Patients who had only received adjuvant chemotherapy were not included. In addition, an Eastern Cooperative Oncology Group performance status score 0–2, a life expectancy >12 weeks, an adequate hepatic, renal and haematological function, and measurable disease by RECIST criteria (Therasse *et al*, 2000) were required.

Exclusion criteria included active second malignancy, prior anti-VEGF therapy, brain metastases, uncontrolled severe infection, major organic failure, ischaemic cardiopathy, bleeding or clotting diatheses and requirement for systemic anticoagulation.

Pretreatment baseline evaluation included a complete medical history, physical examination, full blood count, biochemistry including carcinoembryonic antigen, and a CT scan of the chest, abdomen and pelvis. During treatment, a physical examination and blood cell counts were performed biweekly. Treatment was delayed until recovery in case of neutrophils <1500 mm^–3^, platelets <75 000 mm^–3^ or diarrhoea or stomatitis grade >1 on the planned day of treatment. If treatment had to be delayed for longer than 2 weeks, or any drug discontinued permanently, patients were excluded from the study. Dose reductions of GMB and irinotecan were allowed based upon treatment tolerability. Bevacizumab was withheld in case of gastrointestinal perforation, grade 3–4 haemorrhage, uncontrolled hypertension or arterial thromboembolism.

Maintenance therapy with single-agent bevacizumab was allowed in those patients achieving disease control after six cycles of therapy upon investigators’ discretion.

A refractory disease was defined in those patients in whom progressive disease to the previous line of therapy was documented as best response. Patients who achieved an objective response (complete (CR)/partial responses (PR)) or disease stabilisation (s.d.) but progressed during or within 3 months thereafter from the end of that therapy were considered to have a resistant disease.

Patients’ characteristics and their outcomes were unknown to investigators performing genetic analyses. The local institutional review board approved the study and all patients provided written informed consent before recruitment.

### Study design and treatment

Between January 2005 and October 2008, a total of 89 mCRC patients were enrolled in this two-step study.

### Step 1: Dose-finding study

The dose-finding part of the study was designed considering the previous RD for biweekly GMB within a multidrug regimen in mCRC (Correale *et al*, 2004), the reported lack of a dose–response effect for this agent (Mavroudis *et al*, 2003) and taking into account that CPT-11 efficacy and toxicity are both dose dependent (Abigerges *et al*, 1994). Thus, in the dose-finding part of the study, GMB (1000 mg m^–2^ at a fixed dose rate of 10 mg m^–2^ min^–1^) followed by irinotecan (starting dose of 100 mg m^–2^, with 10 mg m^–2^ increments) and bevacizumab (5 mg kg^–1^) were administered on a biweekly basis. Consecutive cohorts of at least three patients were recruited until the maximum tolerated dose (MTD) was defined. If one out of three patients experienced a DLT, a minimum of three additional patients was enrolled at the same dose level. An MTD was defined if two out of three patients experienced a DLT. The RD was the dose level just below the MTD.

Toxicities were graded according to the National Cancer Institute Common Toxicity Criteria (version 2.0). The DLTs included grade 4 neutropenia lasting >7 days, grade 3–4 neutropenic fever, grade 4 thrombocytopenia, grade 3–4 haemorrhage, grade 3–4 non-haematological toxicity, except for alopecia, nausea or vomiting, gastrointestinal perforation and treatment delay of >4 weeks as a result of toxicity.

### Step 2: Clinical and pharmacogenomic analysis

Once the irinotecan RD was established in a limited dose-finding assessment, this cohort was further expanded in order to prospectively perform an efficacy analysis and an exploratory angiogenesis-directed pharmacogenomic profiling of the combination. Sites of metastatic disease were radiologically re-evaluated every 8 weeks according to standard RECIST criteria unless clinically otherwise indicated (Therasse *et al*, 2000). All responses were independently reviewed and had to be confirmed ⩾28 days after the initial documentation of response. At the time of maximum response, determined by serial CT scans or positron emission tomography if clinically indicated, patients were evaluated by a multidisciplinary team that included surgeons, medical oncologists, hepatologists and interventional radiologists. In this evaluation it was ruled out whether a consolidative approach should be attempted. These approaches consisted of surgical removal of all macroscopic remaining disease, radiofrequency ablation or liver radioembolisation with Yttrium^90^ microspheres.

Serum samples were obtained by centrifugation at 3000 r.p.m. for 10 min and stored at −80 °C until use. Serum levels of VEGF, normalised by the patients platelet count, were determined using a VEGF ELISA (R&D Systems, Minneapolis, MN, USA) according to the manufacturer's instructions.

*VEGF* gene polymorphisms (Supplementary Table 1) were selected if a reported minor allelic frequency >0.20 in a Caucasian population was recorded in the SNP database (http://www.ncbi.nlm.nih.gov/SNP), and/or if the given polymorphism may alter the function of the gene in a biologically relevant manner.

DNA was extracted from EDTA-anticoagulated peripheral blood using the DNAeasy Mini kit (Qiagen, Valencia, CA, USA). Candidate SNPs were genotyped with Taqman-based real-time PCR using the ABI Prism 7900HT Sequence Detection System (Applied Biosystems, Foster City, CA, USA). Primers and probes were obtained from Applied Biosystems as Assays-on-Demand SNP genotyping product (Supplementary Table 1).

### Statistical analysis

Descriptive statistical methodology was used to design and analyse the dose-finding part of the study. Once the RD was achieved, the primary study end point was response rate; secondary end points included characterisation of time to progression (TTP), overall survival (OS) and treatment safety. Analysis of baseline circulating VEGF levels and *VEGF* gene SNPs as predictors of TTP were evaluated at an exploratory level. A two-staged Simon accrual design was adopted with a minimum target activity level (CR+PR) of 20% (Tournigand *et al*, 2004), with the initial stage accruing 24 response-assessable patients. Early discontinuation of the study was planned in the case of less than five responses in the first 24 patients. A minimum planned sample size of 48 evaluable patients was chosen to better estimate efficacy, and a total of 59 patients were recruited. The probability of erroneously concluding that the new treatment is active (*P*⩾0.20) when it is actually ineffective (*P*⩽0.04) is <0.05 (*α*). The probability of erroneously concluding that the treatment is ineffective (*P*<0.20) when is actually effective (*P⩽*0.04) is <0.05 (*β*).

The TTP and OS were calculated from the first day of treatment to the date of first observation of progressive disease or death, respectively. Patients who underwent consolidative procedures after being downstaged with the use of the study regimen were censored at that time for TTP analysis. Patients without documented OS events were censored at last contact. Kaplan–Meier estimates are provided for median TTP and OS, and the log-rank or Breslow tests were applied to test the differences in time-to-event across different genotypes. Differences in circulating VEGF levels were evaluated using the Mann–Whitney *U*-test.

We estimated the false-positive report probability (FPRP) for the observed statistically significant associations using the methods described by Wacholder *et al* (2004). FPRP is the probability of no true association between a genetic variant and a phenotype given a statistically significant finding. It depends not only on the observed *P*-value but also on both the prior probability that the association between the genetic variant and the phenotype is real and the statistical power of the test. In the current study, we set the odds ratio and HR values of 2–4 as a likely threshold value. The prior probability used was 0.25 for all SNPs. The FPRP value for noteworthiness was set at 0.2, which indicates any finding with an FPRP *P*-value of <0.2 is noteworthy.

All statistical tests were performed with the SPSS software v15.0 for Windows (SPSS Inc., Chicago, IL, USA). *P*-values <0.05 were considered statistically significant.

## Results

### Part 1: Dose-finding study

Dose-level (DL) characteristics, the type of DLTs and efficacy results are summarised in Tables 1A and B. In the first five dose levels, no DLT was found. DL6 was expanded to six patients due to one dose-limiting occurrence of grade 3 asthenia. No further DLT were observed. At DL 7 (irinotecan 160 mg m^–2^), two out of three patients developed DLT: grade 3 asthenia (two patients) and grade 3 febrile neutropenia (one patient). The RD was therefore established at DL 6 (150 mg m^–2^). This dose level was subsequently expanded with six more patients, with no further observation of DLT.

### Part 2: Clinical and pharmacogenomic analyses

#### Clinical analysis

Once the RD and preliminary evidence of efficacy were established, 59 additional patients were included in the second part of the trial. Characteristics of these 59 patients are summarised in Supplementary Tables 2 and 3. Most patients (76.3%) received the study regimen as second line. Among them, 28 (62.2%) had received a triplet regimen up-front, (FOLFOXIRI or FOLFOX-Cetuximab), whereas the remaining 17 patients had received first-line therapy with FOLFOX or XELOX. In all, 31 patients (52.5%) treated with the study regimen as second line were considered to have resistant (16 patients) or refractory disease (15 patients). Twenty-one patients received single-agent bevacizumab as maintenance therapy.

The toxicity profile of the combination was mild and it is listed in Supplementary Table 4. The treatment was generally well tolerated in the outpatient setting. The most common grade 3–4 events were haematological. Eight and ten patients had grade 3 leucopenia and grade 3 neutropenia, respectively. Grade 3–4 non-haematological toxicities were rare and included grade 3 diarrhoea and grade 3 asthenia in 5 and 7% of the patients, respectively. Hypertension was the most frequently reported bevacizumab-related toxicity, being of grade 3 in 13.5% of the patients. Other grade 3–4 bevacizumab-specific toxicities included gastrointestinal perforation (3.3%), VTE (1.6%) and bleeding (1.6%) Eleven patients required hospitalisation during the study therapy due to gastrointestinal perforation managed medically (2), haemoptysis (1), neutropenia (5) and fever (3).

On an intent-to-treat basis, overall response rate was 45.7%, with 25 (42.3%) PR and 2 (3.4%) CR. Overall response rate was 63.6 and 30% in patients with sensitive and resistant/refractory disease, respectively (*P*=0.059). Disease control rate (DCR; CR, PR and SD lasting >6 months) was achieved in 32 patients (54.2%). Sixteen patients (27.2%) achieved a sufficient downstaging to undergo a consolidative procedure, including liver surgery (seven patients), thoracic surgery (five patients) or liver radioembolisation with Yttrium^90^ microspheres (four patients).

After a median follow-up of 42 months (range: 10–59), the median TTP and OS were 5.2 (95% CI: 3.4–6.8) and 19.9 months (95% CI: 32–77), respectively.

In the univariate analysis, risk index according to Köhne classification (Kohne *et al*, 2002) and DCR achievement were significantly associated with TTP (Supplementary Table 2). A trend was also found for response to the preceding line of therapy (Supplementary Table 2). In addition, Köhne risk index, response to the previous line of treatment and DCR achievement were all associated to OS (Supplementary Table 2).

#### Serum VEGF levels, VEGF polymorphisms and clinical outcome

Although it remains a controversial issue, standardisation of serum VEGF, normalised by the patients platelet count, has been recommended (George *et al*, 2000), and thus we use this approach in this study.

Baseline VEGF serum levels were significantly higher in Köhne high-risk patients (mean±s.d., 4.42 pg per 10^3^ platelet±2.17 pg per 10^3^ platelet) as compared with the low-risk group (mean±s.d., 2.06 pg per 10^3^ platelet±1.32 pg per 10^3^ platelet) (*P*=0.01).

**Figure 1:**
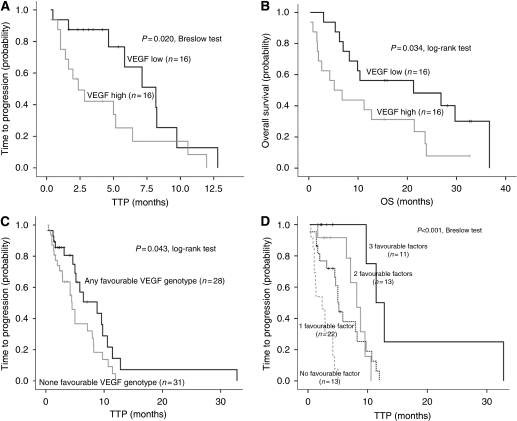
Kaplan–Meier curves for time to progression (TTP) (**A**) and overall survival (OS) (**B**) according to circulating vascular endothelial growth factor (VEGF) serum levels and according to the number of VEGF favourable genotypes (**C**) TTP outcome stratified on basis of the number of favourable clinical and molecular factors (**D**).

VEGF levels were dichotomised into two categories around the median value (platelet-normalised VEGF baseline levels > or <3) to better describe its association with survival times. A significant relationship was found between platelet-normalised VEGF baseline levels and TTP (*P*=0.02; Breslow test) (Figure 1A), with a median TTP of 2.4 (0.83–3.89) months and 8.2 (5.1–11.2) months for patients with high and low VEGF baseline levels, respectively. A significant association was also found between platelet-normalised VEGF baseline levels and OS (*P*=0.034; log-rank test) (Figure 1B), with a median OS of 5.2 (0.27–10.1) months and 21.3 (0.3–47.3) months for patients with high and low VEGF baseline levels, respectively.

As VEGF SNPs contribute to a high variability in VEGF circulating plasma concentrations (Watson *et al*, 2000), we searched for this association in our patients cohort. The VEGF genotype frequencies are shown on Supplementary Table 5. Genotype frequencies of all SNPs followed the Hardy–Weinberg equilibrium. Platelet-normalised serum circulating VEGF baseline levels were significantly lower in VEGF-2578AA and VEGF-460CC carriers (*P*=0.008) and a trend was also observed for VEGF+405GG genotype (Supplementary Figures 1A–C). These results led us to further investigate the correlation between low-VEGF level-associated SNPs and clinical outcome. Patients carrying the VEGF-2578AA genotype had a longer TTP than those with the combined CA and CC genotypes (8.8 *vs* 5 months; *P*=0.080; Supplementary Figure 2A). Similar data were found for the VEGF-460CC genotype (9.6 *vs* 4.5 months; *P*=0.054; Supplementary Figure 2B). There was also a trend for a longer TTP according to VEGF+405 polymorphism (*P*=0.13; Supplementary Figure 2C). Patients harbouring at least one of these genotypes (VEGF-2578 AA, VEGF-460 CC or VEGF+405 GG) showed a significantly longer median TTP than patients possessing none of them (8.8 *vs* 4.5 months; *P*=0.043; Figure 1C).

In an attempt to classify different risk groups in this population, a predictive-risk score was calculated according to the number of favourable clinical (Köhne low-risk category and DCR achievement) and molecular (any favourable VEGF genotype) factors. This analysis rendered four different risk groups, with a median TTP ranging from 2.3 months (95% CI: 0.49–4.25) to 11.4 months (95% CI: 8.5–14.4) corresponding to the groups with none and three favourable factors, respectively (Figure 1D).

In the multivariate model, including the VEGF favourable genotypes and relevant clinical factors according to the univariate analysis (Köhne risk index and DCR), the presence of any favourable VEGF genotype and DCR achievement were both significantly associated with TTP (Table 2). The FPRP was 0.163 for patients carrying any favourable VEGF genotype, indicating noteworthiness.

## Discussion

To our knowledge, this is the first study to report mature results on the combination of GMB, irinotecan and bevacizumab in oxaliplatin/fluoropyrimidines pretreated mCRC. Although it is a heterogeneous group, with most patients receiving the regimen as second line and some as an even later line, if we take into account the type of previous systemic therapies and the high percentage of patients considered to have a truly resistant or refractory disease, our results seem to be encouraging and compare favourably with other irinotecan-based regimens in oxaliplatin-pretreated mCRC (Recchia *et al*, 2004; Tournigand *et al*, 2004; Bidard *et al*, 2009). Furthermore, they overlap with those achieved when bevacizumab is combined with a more active partner than 5-FU (Giantonio *et al*, 2007; Lievre *et al*, 2009) and with other reported GMB-based schedules (Correale *et al*, 2004, 2005; Ziras *et al*, 2006; Lopes *et al*, 2007; Bitossi *et al*, 2008; Merl *et al*, 2009). Although 27% of the patients underwent a consolidative procedure, this finding should be viewed with caution, as the trial was not specifically designed to rule out the resectability rate achieved with this combination.

Dose-limiting toxicities for the combination included grade 3 asthenia and neutropenia. At the RD, toxicity profile was mild. Altogether, 12% of the patients required hospitalisation, but toxicities were uneventfully managed and no toxic deaths were reported. The addition of bevacizumab did not significantly change the side effect profile associated with GMB and CPT-11 (Kakolyris *et al*, 2002; Nishio *et al*, 2005). The low incidence of bevacizumab-specific toxicities encountered in this trial and the previously reported lack of PK/PD interactions between irinotecan and bevacizumab (Denlinger *et al*, 2009) may be partly responsible for these findings. Nevertheless, these results should be viewed with caution, as recent work has suggested that UGT1A1-driven irinotecan dose-escalation studies may more accurately define the precise dosage for this agent (Goetz *et al*, 2007).

To date, most of the tested biomarkers have failed to discriminate patients more likely to benefit from bevacizumab-containing regimens. Assessment of baseline VEGF circulating levels has yielded conflicting results (Burstein *et al*, 2008; Kopetz *et al*, 2010). We initially decided to use serum, instead of plasma, to measure VEGF baseline levels. Plasma VEGF levels are close to the lower limits of detection of the currently available ELISA and, subsequently, serum assessments may provide a greater sensitivity (George *et al*, 2000). Several studies demonstrated that paired serum and plasma VEGF levels correlated in mCRC, and both of them increase with advanced disease stage (Banks *et al*, 1998). In this study, significantly longer median survival times were found in patients with low baseline VEGF levels. Interestingly, low VEGF level-associated SNPs were also correlated with a better clinical outcome in terms of TTP. Similar results were obtained for both, the VEGF-2578A>C and the VEGF-460T>C polymorphisms, in agreement with a previously described strong linkage disequilibrium between both loci. *In vitro* work has also linked the VEGF-2578AA genotype with a decreased VEGF secretion in peripheral blood mononuclear cells (Shahbazi *et al*, 2002) and a lower immunohistochemical VEGF expression in cancer specimens (Schneider *et al*, 2008). Our results show that patients harbouring the VEGF-2578AA genotype achieve an almost two-fold longer TTP compared with alternative genotypes. Similar findings have been reported in metastatic breast cancer patients treated with bevacizumab-based schedules, with VEGF-2578AA carriers showing longer survival times compared with the VEGF-2578CA+CC genotype (Schneider *et al*, 2008). The VEGF-2578CC genotype has also been associated with an inferior median OS compared with alternative genotypes in mCRC patients treated with irinotecan-based chemotherapy and bevacizumab in the first-line setting (Koutras *et al*, 2010).

There may be several potential limitations in these findings. The limited sample size, the exploratory nature of the pharmacogenomic analysis and the complex biological network involved in tumour angiogenesis make it mandatory to confirm these data in larger, prospectively designed clinical trials. Furthermore, Köhne low- and intermediate-risk patients were more likely to have low VEGF baseline levels compared with the high-risk group, and subsequently, a potential confounding interaction between these variables cannot be definitively ruled out. Indeed, VEGF levels have been advocated as a prognostic rather than a predictive factor by other authors (Bernaards *et al*, 2010).

In conclusion, this study suggests that the combination of GMB, irinotecan and bevacizumab may be a valid alternative for oxaliplatin/fluoropyrimidines-pretreated mCRC patients. A further insight into the possible role of *VEGF* gene SNPs as surrogate markers of bevacizumab-based therapy efficacy seems warranted.

## Figures and Tables

**Table 1A:** Irinotecan doses level and DLTs

**Dose (mg m^–2^)**	***N***	**Number of patients with DLT**	**Type of DLT**	**Efficacy**
100–140	15	0	—	2 CR, 5 PR, 6 SD, 2 PD
150	12	1	Grade 3 asthenia.	1 CR, 4 PR, 6 SD, 1 PD
160	3	2	Grade 3 febrile neutropenia Grade 3 asthenia	1 PR, 2 SD

Abbreviations: CR=complete response; DLT=dose-limiting toxicity; PD=progressive disease; PR=partial response; SD=stable disease.

**Table 1B:** Worst-grade toxicity per patient

**Event**	**Level 1–5 (*n*=15)**	**Level 6 (*n*=12)**	**Level 7 (*n*=3)**
*Leucopenia*			
Grade 1–2	5	5	2
Grade 3–4	1	0	1
			
*Neutropenia*			
Grade 1–2	2	2	1
Grade 3–4	0	0	1
			
*Anaemia*			
Grade 1–2	6	2	2
Grade 3–4	1	0	0
			
*Thrombocytopenia*			
Grade 1–2	2	2	0
Grade 3–4	0	0	0
			
*Diarrhoea*			
Grade 1–2	2	4	2
Grade 3–4	0	0	0
			
*Vomiting*			
Grade 1–2	4	5	2
Grade 3–4	0	0	0
			
*Asthenia*			
Grade 1–2	11	3	1
Grade 3–4	0	1	2
			
*Fever*			
Yes	1	0	1
No	14	11	2

**Table 2:** Adjusted Cox multivariate analysis for TTP

**Factor**	**Variable**	**Hazard ratio**	**95% CI**	***P*-value**
Any favourable VEGF genotype	Yes	1.0	—	0.017
	No	2.3	1.1–4.5	—
DCR	Yes	1	—	<0.001
	No	79.4	9.9–630.9	—

Abbreviations: CI=confidence interval; DCR=disease control rate; TTP=time to progression; VEGF=vascular endothelial growth factor.

In the multivariate model, VEGF favourable genotypes and the relevant clinical factors according to the univariate analisis (Köhne risk index and DCR) have been included.
