# Supplementary figures and images for: Dose-finding study and pharmacogenomic analysis of fixed-rate infusion of gemcitabine, irinotecan and bevacizumab in pretreated metastatic colorectal cancer patients
Source: Br J Cancer. 2010 Oct 12;103(10):1529–35. doi: 10.1038/sj.bjc.6605908 (PMC2990573; doi:10.1038/sj.bjc.6605908)

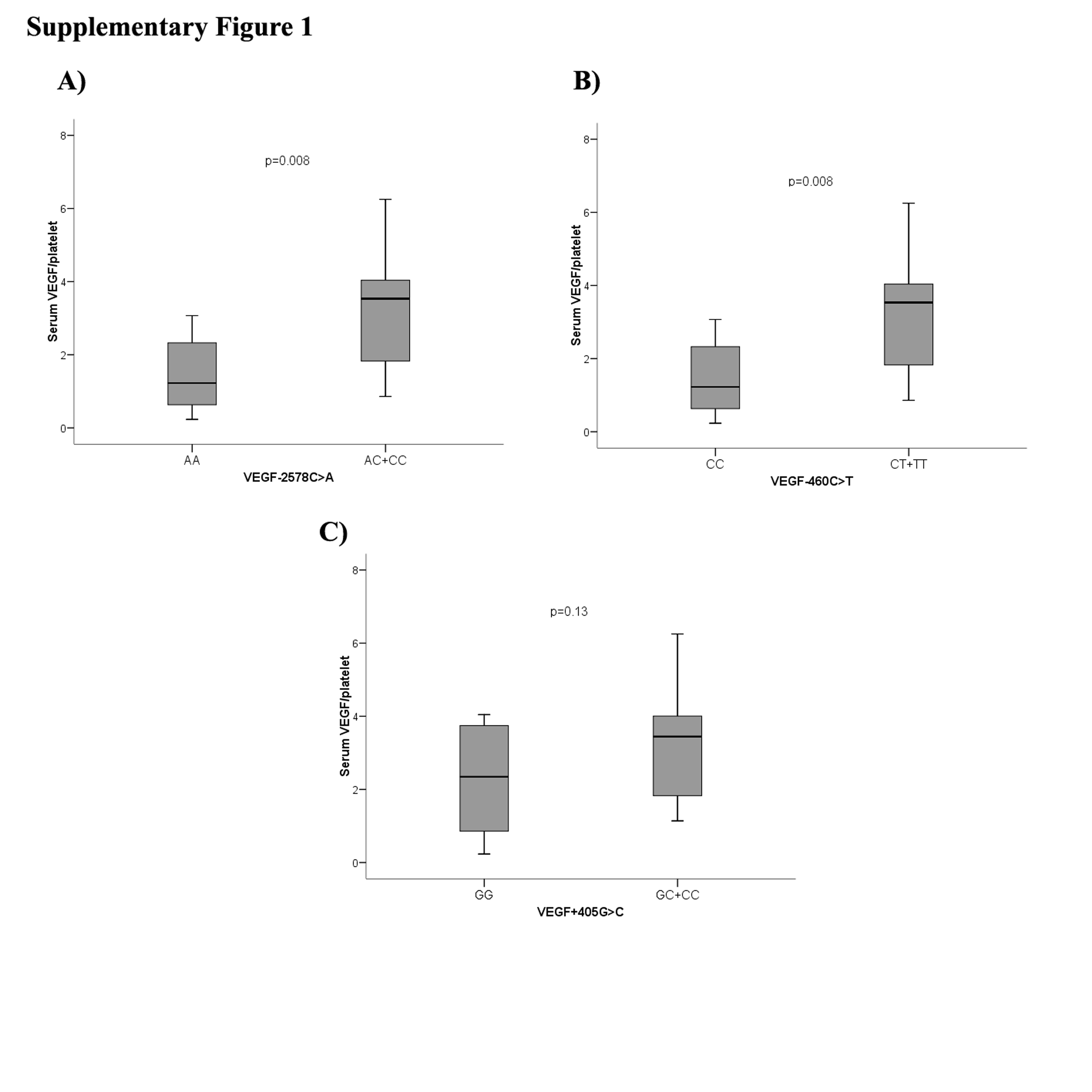

Supplement: Supplementary Figure 1 [file 6605908x1.doc]

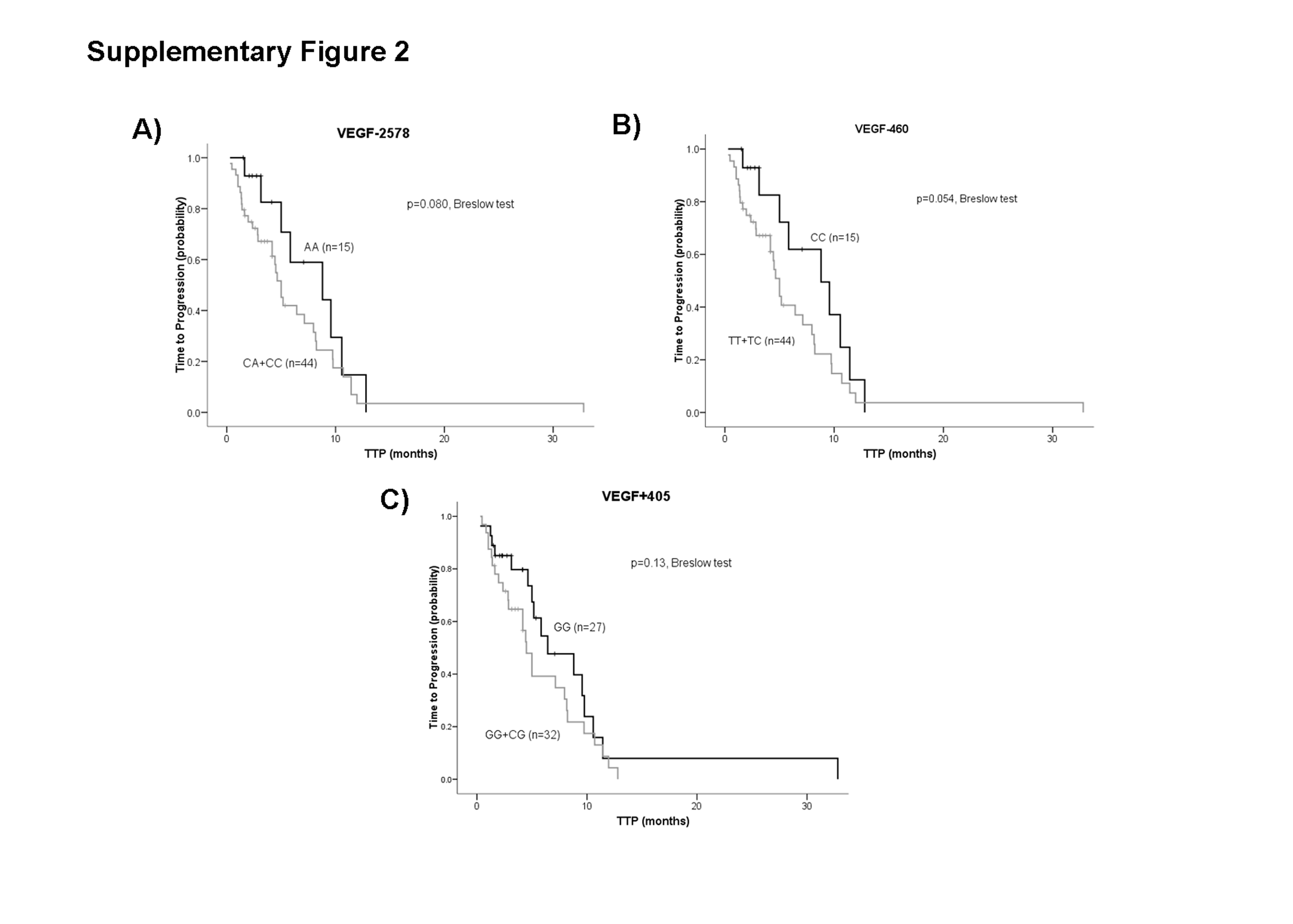

Supplement: Supplementary Figure 2 [file 6605908x2.doc]
