# Supplementary material for: Dose-finding study and pharmacogenomic analysis of fixed-rate infusion of gemcitabine, irinotecan and bevacizumab in pretreated metastatic colorectal cancer patients
Source: Br J Cancer. 2010 Oct 12;103(10):1529–35. doi: 10.1038/sj.bjc.6605908 (PMC2990573; doi:10.1038/sj.bjc.6605908)
Supplement: Supplementary Tables S1–S5 and Figures Legends [file 6605908x3.doc]

**SUPPLEMENTARY TABLES AND FIGURE LEGENDS**

**Supplementary Table 1: Polymorphisms in angiogenesis-related genes selected in this study.**

| **Polymorphism** | **Applied Biosystem Assay ID** | **dbSNP ID** | **Variation** | **SNP location** |
| --- | --- | --- | --- | --- |
| **VEGF-2578C>A** | C_8311602_10 | rs69947 | A/C | Promoter |
| **VEGF-460T>C** | C_1647381_10 | rs833061 | C/T | Promoter |
| **VEGF+405G>C** | C_8311614_10 | rs2010963 | C/G | UTR´5 |
| **VEGF+936 C>T** | C_16198794_10 | rs3025039 | C/T | UTR´3 |
| **VEGF-3´near G>A** | C_1647360_20 | rs10434 | A/G | UTR 3 |

**Supplementary Table 2: Median estimates of TTP and OS considerin**g clinical prognostic factors.

|  |  | **TTP** | | **OS** | |
| --- | --- | --- | --- | --- | --- |
| **Variables** | **(n, %)** | **Median (95% CI)** | **p ^a^** | **Median (95% CI)** | **p ^a^** |
| **Age** |  |  |  |  |  |
| <60 | 29 (49%) | 7.9 (4.7-11.1) | 0.190 | 21 (8.3-34.2) | 0.500 |
| ≥60 | 30 (51%) | 4.4 (2.9-5.9) |  | 10.4 (7.7-13.1) |  |
| **Sex** |  |  |  |  |  |
| Male | 38 (64.4%) | 5.1 (4.4-5.9) | 0.93 | 11.2 (0.1-25.1) | 0.428 |
| Female | 21 (35.5%) | 5.8 (0.9-10.7) |  | 19.9 (8.9-30.9) |  |
| **Köhne risk index** |  |  |  |  |  |
| Low | 21 (35.6%) | 8.1 (4.6-11.7) | 0.001 | 29.7 (23.5-35.9) | <0.001 |
| Intermediate risk | 24 (40.1%) | 6.4 (2.5-10.4) |  | 10.4 (6.9-13.9) |  |
| High risk | 14 (23.7%) | 2.8 (0.1-5.6) |  | 5.2 (3.9-6.4) |  |
| **Prior chemotherapy treatment** |  |  |  |  |  |
| 1 lines | 45 (76.3%) | 5 (3.4-6.5) | 0.215 | 11.8 (0.1-27.8) | 0.749 |
| ≥2 lines | 14 (23.7%) | 7.9 (3.2-12.6) |  | 21.2 (7.7-34.8) |  |
| **Response to previous lines of therapy** |  |  |  |  |  |
| sensitive | 28 (47.4%) | 7.1 (3.8-10.4) | .054 | 23.6 (16.5-30.7) | 0.010 |
| refractory/resistant | 31 (52.5%) | 4.4 (2.2-6.6) |  | 8.9 (4.9-12.9) |  |
| **DCR achieved (no/yes)** |  |  |  |  |  |
| no | 27 (45.7%) | 2.8 (1.3-4.3) | <0.001 | 5.3 (3.8-6.8) | <0.001 |
| yes | 32 (54.2%) | 9.7 (8.5-11.1) |  | 28.2 (22.7-33.7) |  |

Kaplan Meier estimates of TTP and OS. **^a^** Difference of the estimates tested using the log-rank or Breslow test.

**Supplementary Table 3: Previous systemic therapies.**

| **Global population (n=59)** | **n** | **%** |
| --- | --- | --- |
| Oxaliplatin | 59 | 100 |
| Fluoropyrimidines | 59 | 100 |
| Irinotecan | 27 | 45.8 |
| Cetuximab | 18 | 30.5 |
| **Patients treated as second-line (n=45)** |  |  |
| Schedule | **Resistant (n)** | **Refractory (n)** |
| FOLFOXIRI | 1 | 1 |
| FOLFOX-Cetuximab | 7 | 5 |
| FOLFOX/XELOX | 8 | 9 |

**Supplementary Table 4:** Toxicity profile of gemcitabine, irinotecan and bevacizumab.

VTE; Venous thromboembolism

| **Adverse event** | **Grade 1 (n, %)** | **Grade 2 (n, %)** | **Grade 3 (n, %)** | **Grade 4 (n, %)** |
| --- | --- | --- | --- | --- |
| Leucopenia | 17 (29) | 6 (10) | 8 (14) | 0 (0) |
| Neutropenia | 5 (8) | 7 (12) | 10 (17) | 0 (0) |
| Anaemia | 33 (56) | 9 (15) | 2 (3) | 0 (0) |
| Thrombocytopenia | 2 (3) | 4 (7) | 0 (0) | 1 (2) |
| Vomiting | 10 (17) | 6 (10) | 0 (0) | 0 (0) |
| Diarrhoea | 9 (15) | 17 (29) | 3 (5) | 0 (0) |
| Mucositis | 6 (10) | 2 (3) | 0 (0) | 0 (0) |
| Asthenia | 10 (17) | 32 (52) | 4 (7) | 0 (0) |
| GI perforation | 0 (0) | 0 (0) | 2 (3.3) | 0 (0) |
| Hypertension | 6 (10.1) | 4 (6.7) | 8 (13.5) | 0 (0) |
| VTE | 1 (1.6) | 0 (0) | 1 (1.6) | 0 (0) |
| Bleeding | 5 (8.4) | 3 (5) | 1 (1.6) | 0 (0) |

**Supplementary Table 5:** Allele and genotype distributions of the polymorphisms in the VEGF gene: association with TTP and OS.

| **Polymorphism/ genotype** | **N (%)** | **TTP (median)** | **p^a^** | **OS (median)** | **p^a^** |
| --- | --- | --- | --- | --- | --- |
| **VEGF-2578C>A** |  |  |  |  |  |
| C/C+C/A | 44 (74.6%) | 5 (4.2-5.7) | 0.08 | 10.4 (0.1-22.1) | 0.120 |
| A/A | 15 (25.4%) | 8.8 (1.8-15.8) |  | 25.9 (16-35.8) |  |
| **VEGF-460T>C** |  |  |  |  |  |
| C/C | 44 (74.6%) | 8.8 (3.9-13.7) | 0.054 | 25.9 (6.7-45.2) | 0.117 |
| T/T+T/C | 15 (25.4%) | 5 (4.3-5.7) |  | 10.4 (0.1-22.1) |  |
| **VEGF+405G>C** |  |  |  |  |  |
| G/G | 27 (45.7%) | 6.4 (2.4-10.5) | 0.138 | 21.4 (3.7-39.1) | 0.291 |
| G/C+C/C | 32 (54.3%) | 4.5 (3.6-5.4) |  | 10.4 (0-24.5) |  |
| **VEGF+936 C>T** |  |  |  |  |  |
| C/C | 39 (69.6%) | 6.4 (2.2-10.9) | 0.333 | 23.6 (18.9-28.2) | 0.207 |
| C/T | 14 (25%) | 4.4 (3.0-5.8) |  | 8.9 (3.7-14) |  |
| T/T | 3 (5.4%) | 5.8 (0.1-13.2) |  | 6.2 (1.1-11.3) |  |
| **VEGF-3´nearG>A** |  |  |  |  |  |
| G/G | 23 (40.4%) | 6.4 (3.8-9.0) | 0.988 | 11.7 (4.9-18.6) | 0.988 |
| G/A | 26 (45.6%) | 5 (1.5-8.4) |  | 19.9 (4.8-35.0) |  |
| A/A | 8 (14%) | 5.1 (0.07-10.2) |  | 26.8 (10.9-42.7) |  |

Kaplan Meier estimates of TTP and OS. **^a^** Difference of the estimates tested using the log-rank or Breslow test.

**SUPPLEMENTARY FIGURE LEGENDS:**

**Supplementary Figure 1:**

Platelet-normalized serum circulating VEGF levels according to the different VEGF genotypes. VEGF-2578AA and VEGF-460CC carriers showed significantly lower VEGF levels (A, B) and a trend was observed for VEGF+405GG genotype (C).

**Supplementary Figure 2:**

Kaplan-Meier curves for the TTP outcomes according to the different VEGF genotypes. VEGF-2578AA (A) and VEGF-460CC (B) genotypes carriers showed a longer median TTP. A trend was also observed in patients with VEGF+405GG (C).
